# Role of Caveolin-1 in Sepsis – A Mini-Review

**DOI:** 10.3389/fimmu.2022.902907

**Published:** 2022-07-15

**Authors:** Pamella Silva Lannes-Costa, Bruna Alves da Silva Pimentel, Prescilla Emy Nagao

**Affiliations:** Laboratory of Molecular Biology and Physiology of Streptococci, Institute of Biology Roberto Alcantara Gomes, Rio de Janeiro State University (UERJ), Rio de Janeiro, Brazil

**Keywords:** caveolin 1, sepsis, cellular permeability, apoptosis, autophagy

## Abstract

Sepsis is a generalized disease characterized by an extreme response to a severe infection. Moreover, challenges remain in the diagnosis, treatment and management of septic patients. In this mini-review we demonstrate developments on cellular pathogenesis and the role of Caveolin-1 (Cav-1) in sepsis. Studies have shown that Cav-1 has a significant role in sepsis through the regulation of membrane traffic and intracellular signaling pathways. In addition, activation of apoptosis/autophagy is considered relevant for the progression and development of sepsis. However, how Cav-1 is involved in sepsis remains unclear, and the precise mechanisms need to be further investigated. Finally, the role of Cav-1 in altering cell permeability during inflammation, in sepsis caused by microorganisms, apoptosis/autophagy activation and new therapies under study are discussed in this mini-review.

## Introduction

Sepsis is one of the main causes of morbidity and mortality in patients admitted to the intensive care unit, generating high socioeconomic impacts for health systems around the world. Sepsis affects between 200 and 1,000 patients per 100,000 inhabitants/year, being a condition with a high risk of death associated with an impaired immune response during systemic infection. Currently, therapies used in the treatment of sepsis have been failing to improve clinical outcomes. The identification of molecules involved in sepsis as modulators of the immune response, cell recognition and signal transduction are crucial for a better understanding of the disease, providing new insights for more efficient treatment (1–4).

Caveolae are invaginations in the plasma membrane, composed of lipids, rich in cholesterol and sphingolipids expressed in various cell types. Caveolae participate in the regulation of lipid and glucose metabolism, immunocyte maturation, and inflammatory responses, in addition to signaling molecules such as nitric oxide synthase (NOS), Toll-like receptors (TLRs), MAPK cascade components and Src family tyrosine kinases, which provide a platform for signal transduction (5–7).

Caveolin-1 (Cav-1) is a major and essential structural protein of caveolae, used as a marker molecule for caveolae. Deletion of the Cav-1 gene led to the loss of caveolae, demonstrating the crucial role of Cav-1 in the formation of caveolar microdomains. Cav-1 appears to act as a scaffolding protein able to recruit and modulate the activity of caveolae-localized signalling molecules (8, 9). Due to the important role of caveolae in signal transduction and based on evidence on the protective role of Cav-1 in the inflammatory response, the present review will focus on the role of Cav-1 during sepsis.

## Caveolin-1 (Cav-1)

Cav-1 was initially recognized as a phosphoprotein substrate of Src kinase that afterwards was described as a major structural component of the caveolae membrane *in vivo* (10). The Cav-1 scaffold has been poorly described in comparison to caveolae and planar rafts. Oligomerization of Cav-1 regulates EGFR activity independently of caveolae formation and defines a domain distinct from caveolae (11). Cav-1 is expressed in different cell types, including adipocytes, endothelial cells and fibroblasts; in addition to multiple organelles such as intracellular vesicles and the Golgi apparatus (10). Cav-1 is important during establishing specific lipid rafts and helps compartmentalize signal pathways, including cell cycle regulation, endocytosis, cholesterol trafficking and efflux (12–14).

The coordinated involvement of Cav-1 with actin cytoskeleton dynamics contributes to mechanosensitization and adaptation to mechanical stimuli to environmental changes such as microbial infection (15). Formin proteins associated with actin filaments have been described during maintenance and organization of the dynamics of cytoplasmic Cav-1, mainly to mechanical challenge (16). Thus, Cav-1 regulates several signaling pathways, promoting crosstalk between molecules, where alterations and/or disturbances in the positioning of Cav-1 in the plasma membrane can affect cell signaling and biological crosstalk (10, 17).

### Cav-1 and Multiple Signalling Receptors/Molecules

The functional dysregulation or protein abundance of Cav-1 leads to numerous pathophysiological processes, which are risk factors for the development of diseases. Cav-1 interacts with signaling molecules located in the caveolae, such as G-protein-coupled receptors, Src family molecules, receptor tyrosine kinases, type 1 angiotensin II receptor, ion channels and endothelial nitric oxide synthase (eNOS), a critical enzyme involved in maintaining endothelial cell homeostasis *via* nitric oxide production (18, 19).

Inflammatory cytokines play a central role in the self-defense system against microbial pathogens (20). Furthermore, an excessive activation of the inflammatory system results in overload of endogenous antioxidant production - reactive oxygen species (ROS) and nitrogen; promoting impacts on cell signaling systems based on redox potential, direct damage to biomolecules and immunosuppression. Excessive ROS have been associated with several stages of sepsis in both preclinical and clinical studies. Cav-1 can directly regulate NADPH oxidase-induced ROS production (21, 22), modulating *in vivo* oxidative stress and inflammatory responses to LPS challenge (22, 23).

Activation of Rac1 by Cav-1 involves NADPH oxidase assembly. The Rac1 and NADPH oxidase subunits have been shown to localize to caveolae containing Cav-1 in various cell types (24). Activation of Rac1 and Rac2 did not occured in Cav-1^-^/^-^ polymorphonuclear neutrophils (PMN). Thus, PMN-expressed Cav-1 played an important role in the mechanism of inflammation and lung injury responses mediated by PMN activation (25). Additionally, lymphocytes have a critical function in the adaptive immune system. They recognize pathogenic signals through T-cell and B-cell antigen receptors to be activated and perform their protective function. However, inappropriate activation of T and B cells can be harmful to the host, resulting in autoimmune disorders and immunodeficiencies. Although Cav-1 is expressed at low levels in primary lymphocytes, studies have demonstrated the importance of Cav-1 in the organization of the basement membrane of T cell and B cell antigen receptors, as well as its reorganization after activation (–19). Previous publication has shown that Cav–1 ablation promoted T cell receptor phosphorylation and downstream signaling in CD4+ T cells (26). The Cav–1 deletion altered actin polymerization after T cell receptor activation, decreasing the traffic of lipid rafts to the contact region between CD8+ T cells and target cells (27). In both T cell subpopulations, Cav–1 controled lipid membrane reorganization following T cell receptor activation such as the coalescence of the SRC kinase LCK family and T cell receptor nanoclusters in CD4+ T cells, and actin filaments and lipid raft junction in CD8+ cells. In this way, Cav–1 regulated the proximity between the T cell receptor and its activators/effectors, through the actin cytoskeleton, regulating membrane dynamics and compartmentalization after T cell receptor activation (19).

The recruitment of TLR4 into lipid rafts has been observed upon LPS stimulation. Cav–1 is thought to play a central role in signal regulation within the caveolae through direct regulatory interactions with TLR4. Cav–1 and TLR4 may act together on the hypoxic trophoblast–induced permeability of the endothelial cell monolayer (28). Cav–1 regulated the expression of mediators that govern LPS–induced inflammatory signaling in mice, and deletion of Cav–1 suppressed the inflammatory response mediated by the LPS–CD14–TLR4–NF–κB pathway, alleviating acute injury in mice (29). In addition, in silico modeling revealed the potential of the toxin produced by fungal species to intercalate into caveolae, especially the interaction of Cav–1 with the TLR 4 during activation of the inflammatory response (30).

The regulatory roles of Cav–1 in immune cells range from its regulator of signal transduction to endocytosis. TGF–β has been shown to regulate the activation of the M2 phenotype in macrophages (31). As Cav–1 regulates TGF–β signaling, it is rational to suggest the role of Cav–1 in the maturation and differentiation of immune cells, mainly the subtype M2 macrophages (32). Relevant for airway diseases, Cav–1 has been shown to be the costimulatory ligand for CD26, resulting in strong T cell costimulation and NF–κB activation in a T–cell receptor/CD3–dependent manner (33). Cav–1 has also been shown to upregulate monocyte differentiation into macrophages through of the the early growth response–1 transcription factor and confers anti–inflammatory effects on macrophages (34).

The functions of Cav–1 during cell regulation may have important implications during sepsis. Cav–1–deficient mice were more susceptible to death by polymicrobial sepsis when compared to the wild–type mice, exhibiting distinct inflammatory responses. Wild–type mice exhibited rapid induction of TNF–α and IL–6 in the early stage of sepsis and a significant decrease in TNF–α and IL–6 levels thereafter and increased production of antiinflammatory IL–10 cytokine in the LPS model. In contrast, Cav–1 nulls exhibited exaggerated generation of inflammatory cytokines during sepsis, demonstrating extended and uncontrolled cytokine production in the absence of Cav–1 (35). Recent publication showed CD39 co–localized with Cav–1 on LPS–treated macrophages, and that P2X7 receptor inhibition promoted inhibition of CD39/Cav–1 co–localization. Furthermore, STAT3 activation was attenuated in Cav–1–deficient macrophages treated with LPS, suggesting that P2X7 receptor trigger early lipid raft–dependent mechanisms that upregulate CD39 activity and contribute to limiting macrophage responses (36). Other studies showed that Cav1 coordinated and coupled with Notch1/HES1 in the liver tissue of LPS–induced septic mice, which promoted the production of IL–10 in macrophages, leading to inhibition of inflammation in a model of sepsis (3).

**Figure 1:**
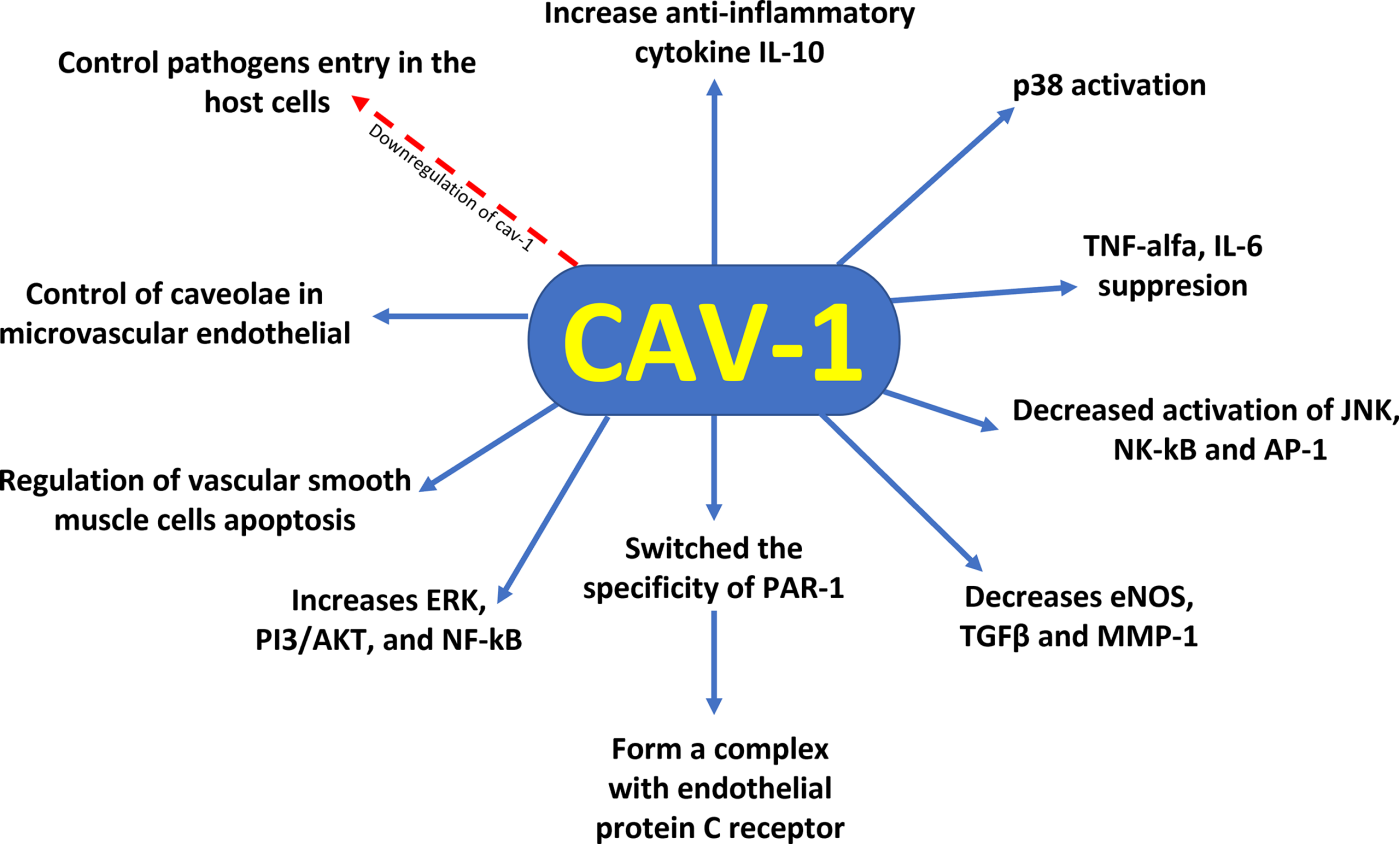
Cytoprotective profile of Cav–1 against sepsis–induced mortality. Downregulation of caveolin–1 decreases caveolae available to control pathogen entry into host cells, control of caveolae in the microvascular endothelia of the lung reveals regulation of vascular smooth muscle cells apoptosis, reduction of Cav–1 switches the specificity of PAR–1 to initiate protective response in endothelial cells by altering the occupancy of endothelial protein C receptor. In addition, Cav–1 has a protective role for inflammation by suppression of proinflammatory cytokines (TNF–α and IL–6) production, augmentation of antiinflammatory IL–10 cytokine production, and involvement of the p38 MAPK, JNK, NK–κB, and AP–1 signal pathways.

Moreover, evidence showed that the functions of Cav–1 involved the p38 MAPK kinase signalling pathway. The increased activation of p38 by LPS and decreased activation of JNK, NK–κB, and AP–1 by Cav–1 lead to protection against inflammation (37). Furthermore, protein C administration during sepsis resulted in decreased levels of inflammatory cytokines, apoptosis and Cav–1. Decreased Cav–1 expression promoted changes in protease–activated receptor–1 (PAR–1) specificity to initiate protective response in endothelial cells, altering endothelial protein C receptor (EPCR) occupancy (38) (**Figure 1**). Thus, the modulation of Cav–1 expression through different cytoprotective mechanisms can modify lung function at both cellular and systemic levels. The differential regulatory roles of Cav–1 for different receptors and intracellular signal transduction pathways demonstrate the complex involvement of Cav–1 in the development of diseases, making Cav–1 a candidate in the battle against sepsis–induced mortality.

### Cav–1 in the Lung

The lung is an organ that has direct contact with the external environment, serving as a crucial immune organ, which harbors innate and adaptive immune cells to induce a potent immune response. Due to its direct contact with the external environment, the lung serves as a primary target organ for many pathogens that cause pneumonia, acute respiratory distress syndrome, acute lung injury or inflammation and sepsis. The potent generation of the innate immune system response in the lungs during infections or its dysregulation, as seen in non–pulmonary sepsis, plays a crucial role in the outcome of the disease (39).

The pulmonary innate immune response during infections initiates with the activation of residential innate immune cells (airway epithelial cells, macrophages, dendritic cells and innate lymphoid cells) inducing the neutrophil infiltration into the lungs. Alveolar epithelial cells (pneumocytes type I primarily involved in facilitating gaseous exchange and may recognize pathogens and pneumocytes type II that serve as innate immune cells) serve as a protective mechanical barrier against inhaled pathogens responsible for diseases. Thus, the pulmonary immune system is separable into compartments, which Cav–1 have a significant role during respiratory tract infection (40). Cav–1 plays an important role in the function and homeostasis of the lungs after birth. Studies have shown that in the lung Cav–1 is highly expressed in airway smooth muscle, alveolar (mainly in type I pneumocytes) and bronchial epithelium, lung fibroblasts, inflammatory cells, pulmonary vasculature, particularly in arterial smooth muscle and endothelium (41). The widespread presence of Cav–1 increases controllability and may influence the pathophysiology in severe lung disease states. In addition, Cav–1 colocalizes with tight junction (TJ) proteins in pulmonary epithelial cell and it negatively regulates inter–endothelial junctional permeability (42).

Cav–1 phosphorylation was required for interaction with TLR4 and activation of TLR4–MyD88 signaling and sepsis–induced lung inflammation. Inhibition of Cav–1 Tyr14 phosphorylation and the resulting inactivation of TLR4 signaling in pulmonary vascular endothelial cells represented a strategy to prevent sepsis–induced lung inflammation and injury (43). Furthermore, relevant to lung function, Cav–1 modulated intracellular Ca2+ and contractility of airway smooth muscle. Although studies in mice Cav–1 KO provide important information about protein function, they do not discriminate between the various types of lung cells and therefore many questions about the role of Cav–1 remain unanswered (44). Thus, searching for Cav–1 mutations and polymorphisms may be important in determining the roles of Cav–1 in the pathogenesis of lung disease.

Data from *in vitro*, animal and human studies conducted in recent years have revealed that Cav–1 regulates microvascular permeability, contributes to PMN neutrophil–mediated acute injury, and mediates alveolar cell death during lung injury (45). Cav–1 acts as a facilitator of inflammatory effects in the airways. However, studies have demonstrated pleiotropic effects of Cav–1 on airway structure vs. function that may be dependent on the cell and the analyzed context. Up and down regulation of Cav–1 can contribute to lung disease through several mechanisms. For example, factors such as pro–inflammatory cytokines and cholesterol can increase Cav–1 expression while other signaling intermediates suppress Cav–1 expression. Downstream of Cav–1 can inhibit eNOS and extracellular components such as TGF–β and MMP1, but increases pro–inflammatory signaling *via* ERKs, PI3/Akt or NF–kB (46) (**Figure 1**). In lung fibrosis, Cav–1 downregulation in the parenchyma is associated with downstream activation of TGF–β pathway, aberrant extracellular matrix production and parenchymal remodeling (47). Furthermore, this differential immunopathogenesis mechanism triggered by oxidative stress and signaled by Cav–1can lead to heterogeneous lung pathologies (48).

### Cav–1 in Brain

Exacerbated peripheral inflammation and reactive oxygen species can induce neuroinflammation with severe central nervous system (CNS) impairment. Sepsis–associated disruption of the blood–brain barrier promotes a pro–inflammatory cytokine storm in the CNS that leads to brain dysfunction in sepsis survivors (49).

Brain endothelial cells (BECs) serve at the frontline, facing the bloodstream and thus, play a dominant role in determining blood–brain barrier permeability. BECs control paracellular and transcellular passage pathways through two unique features: increased expression of TJ proteins to preclude paracellular passage of blood–borne molecules and cells, and restricted transcytosis and fenestration to restrain non–specific transcellular transport of blood contents. In the CNS endothelium, most endocytic vesicles are non–clathrin–coated caveolae containing Cav–1. During inflammation, immune cells cross blood vessels of multiple organs to mount an appropriate immune response. Immune cells can extravasate through either TJs (paracellular migration) or endothelial vesicles (transcellular migration). In neuroinflammation, caveolae–independent TJ remodeling facilitates Th17 lymphocyte transmigration across the BBB, whereas Cav–1 regulate the entry of Th1 lymphocytes into the CNS. Loss of Cav–1 selectively reduces Th1 cell infiltration into the CNS (50).

Although barrier disruption promotes neuroinflammation by enabling inflammatory proteins and leukocytes to access the CNS, how dynamic remodeling of TJs correlates with disease pathogenesis and its importance for immune cell trafficking remains unclear.

*In vitro* cell studies reveal that BBB breakdown is partially caused by Cav–1–mediated redistribution of membranous claudin 5 into the cytosol under hypoxia (51). The down regulation of Cav–1 in vascular endothelial cells by oxidative stress activates anti–apoptotic pathways and endothelial proliferation. Several mediators of Ca2+ signaling have been found to be associated with caveolae and Cav–1 deficiency in endothelial cells has been shown to impair plasma membrane Ca2+ entry (52). All these pathways have important implications for diverse processes in brain endotelial cells including the response to shear/mechanical stress, cellular proliferation/migration, regulation of vascular permeability/tone and sepsis.

## Cav–1 and Barrier Permeability

Functional alterations of endothelial cells in sepsis such as vasoregulation, alteration of the cell barrier, hemostasis and inflammation are considered essential in the progression of sepsis to multiple organ failure. Activation and recruitment of immune cells to sites of inflammation are necessary in combating exogenous microorganisms (53). Moreover, this mechanism can lead to injury to the living tissue if leukocyte recruitment is not adequately controlled. Additionally, alteration of vascular endothelial permeability induces excessive fluid loss from the intravascular space, causing vascular hypotension. Monitoring endothelial cell line function can be considered the main obstacle in the evaluation of endothelial dysfunction, as well as the therapy to be used in critical illness states of sepsis (54). Excessive increase in vascular permeability causes severe intravascular hypovolemia and hypotonia that are still challenge in the sepsis treatment. Volume resuscitation and use of vasopressors can cause unwanted complications, being associated with increased organ dysfunction and mortality (55). Diagnostic methods to assess endothelial cell function during sepsis will have to be synchronized with interventions to treat endothelial tissue at risk during sepsis. Caveolae control membrane tension and respond to fow, shear stress and cell stretch (56). Cav–1 regulates microvascular permeability, contributes to neutrophil–mediated acute injury, and mediates alveolar cell death during lung injury (45).

A recent study demonstrated that Cav–1 plays a critical role in the regulation of endothelial transcellular permeability (57). Upregulation of Cav–1 has been associated with resistance to anoikis, resulting in loss of adhesion to the cell matrix. The depletion of c–Src inhibited the induced damage of the microvascular endothelial barrier, regulating Cav–1 phosphorylation and caveolae formation (58).

Acute lung injury is a disorder of sepsis, characterized by pulmonary edema and inflammatory infiltrate that can lead to disruption of TJ, resulting in disruption of pulmonary epithelial and endothelial barriers. Increased pulmonary microvascular permeability will cause pulmonary edema (59). TJ are barriers between epithelial and endothelial cells controlling paracellular permeability. Occludin, claudins and zonula occludens are the main members of TJ proteins that contribute to the diffusion barrier and permeability resistance (60). TJ barrier dysfunction is correlated with changes in TJ lipid rafts during polymicrobial sepsis. Downregulation or mislocalization of TJ proteins is known to result in destruction of the pulmonary epithelial barrier.

A previous study showed that Cav–1 acts on the endocytosis of occludin during vascular permeability. Endocytosis of TJ proteins from the cell membrane, intracellular transport vesicles, recycling and degradation are regulatory mechanisms for plasticity and barrier properties (61). Furthermore, Cav–1 appears to be involved in occludin recycling in human brain endothelial cells. Cav–1–associated claudin–5 redistribution was critical in the late phase of BBB breakdown. Cav–1–mediated internalization of claudin–5 resulted in the elimination of claudin–5 from human brain endothelial cell membranes during paracellular opening of BBB (57). Additionally, Cav–1 overexpression attenuated BBB disruption and consequent extracellular brain edema through the inhibition of TJ protein degradation, suggestting a high relationship between TJ and Cav–1 (62).

The population of endothelial cells shows significant diversity, including physiological differences between macrovasculature, microvasculature or lymphatics, as well as differences that have not yet been explored. Thus, although Cav–1 is expressed throughout the pulmonary vascular network, their expression may vary from vessel to vessel and between subpopulations, and may represent an importante factor capable of regulating resting to activated endothelial cell phenotype (63). The adherens junction (AJ) mainly consists of VE–cadherin binding to VE–cadherin of neighboring endotelial cells and catenins linking VE–cadherin to the actin cytoskeleton. Evidence was obtained for the phosphorylation state of VE–cadherin in maintaining functional epitelial and endotelial junctions. Changes in VE–Cadherin junctional expression can also have profound impact on barrier function, and Cav–1 has been reported to be required for AJ assembly (64).

Mechanisms underlying endothelial barrier function loss include eNOS/NO/ROS/NADPH Oxidase signaling and tyrosine kinase signaling (65). Moreover, nucleoside diphosphate kinase B contributes to the regulation of AJ integrity through interaction with Cav–1 (66). Experiments demonstrated that vascular endothelial growth factor (VEGF) – VEGF receptor type 2 (VEGFR–2) induced the phosphorylation of Cav–1 by c–Src, necessary for the opening of the AJ (67). Another study showed that phosphorylation of Cav–1 on Tyr14 was necessary for thrombin–induced AJ opening (68).

The mechanisms by which Cav–1 protects against the breakdown of BBB permeability may be involved in the regulation of signalling components. Overexpression of Cav–1 reduced eNOS activity, which can be blocked by NO production, in addition, Cav–1 KO mouse model protected against LPS–induced pulmonary hyperpermeability and edema production through increased eNOS activity (17, 59, 69). Moreover, reduction of Cav–1 expression increased the barrier permeability through the upregulation of metalloproteinases (MMPs) in endothelial cells (70). The Cav–1 inhibited MMP–2 activity in a dose–dependent manner (71). The stability of cell–to–cell contacts formed by TJ and AJ depends on the connection with actin filaments. However, when the cytoskeleton dynamics is disturbed, it can alter the balance between cortical actin and contractile stress fibers, promoting cell barrier dysfunction and consequent increase in vascular permeability (62). Altered microfilament dynamics may favor the over– recruitment of immune cells to inflamed tissue. Modifications in microfilament formation play a role in cell barrier regulation and vascular permeability. The integrity of endotelial tissue requires a fine balance between the formation of cortical microfilaments that promote the stability of cell–cell contacts and the formation of actin stress fibers that are responsible for the tensile strenght and contact stability of endothelial cells (55). The researchers analyzed the importance of Cav–1 in mechanosensitization and cellular mechanoprotection, demonstrating that the dynamics of cytoplasmic Cav–1 was strongly associated with microfilaments (72).

The research aims to improve the understanding of pathophysiological pathways that control cell barrier dysfunction and increased vascular permeability to implement new therapeutic strategies for the treatment of sepsis. Furthermore, modifying microfilaments dynamics in human endothelial cells could be na important tool for the development of treatment options for sepsis (73). Currently, sepsis treatment does not address the underlying molecular pathogenesis, but only attempts to reduce the harmful consequences of sepsis.

## Cav–1 Play a Critical Role During Sepsis by Pathogens

Caveolar microdomains have been implicated in the regulation of signal transduction associated with host infection by various microorganisms, including sepsis–causing pathogens. Cav–1 had a significant protective role in sepsis and endotoxemia (74). However, most experimental assays have used cholesterol–sequestering drugs that do not distinguish caveolae from other molecules that form lipid rafts (1, 2, 73, 74). In this way, more specific analyzes such as overexpression, small interfering RNA (siRNA) and knockout (KO) animals can provide more accurate results. The use of KO mice has allowed a more significant analysis of Cav–1 in the regulation of cell signaling in different models of sepsis.

**Figure 2:**
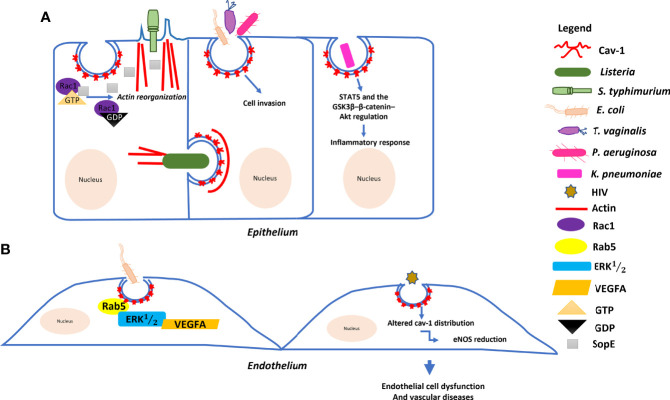
Model of the molecular mechanism underlying the role of Cav–1 in bacterial and HIV invasion: **(A)** *Salmonella typhimurium* delivers SopE effector proteins into the host cell through a type III secretion system. SopE activates Rac1 leading to the activation of actin reorganization. Cav–1 interacts directly with these proteins to form a complex, enhancing *Salmonella* invasion into host cells. *Listeria monocytogenes* can trigger Cav–1 endocytosis for cell–to–cell transfer involving actin filaments. *Pseudomonas aeruginosa*, *Escherichia coli* and *Trichomonas vaginalis* empregues Cav–1–mediated endocytosis to invade host cells. *Klebsiella pneumoniae* affects both systemic and local production of proinflammatory cytokines *via* the actions of STAT5 and the GSK3β–β–catenin–Akt pathway. **(B)** Endocytosis of *E. coli* is regulated by the small GTPase Rab5 through interaction with Cav–1 and plays multiple roles in the VEGFA–induced signaling cascade to enhance permeability in human endothelial cells. HIV also induces endothelial cell dysfunctions through Cav–1.

Pathogenic microorganisms, such as those involved in sepsis, choose caveolae as a gateway to invade host cells, grow and replicate in an intracellular environment without lysosomal degradation. Furthermore, a host mechanism has been observed to decrease intracellular bacterial load through decreased expression of the Cav–1 protein, removing caveolae available for endocytosis. The invasion of human cells by *Campylobacter jejuni* was inhibited by lipid rafts disruptors (75). Currently, the ability of microorganisms to adhere to caveolae and/or receptors located in caveolae to enter and move into host cells has been reported. Some examples of the role of Cav–1 during sepsis induced by pathogenic microorganisms are shown in **Table 1** and **Figure 2**.

**Table 1:** Role of Cav-1 during sepsis by pathogens.

MICROORGANISM/ LPS or CLP MODEL	ANIMAL OR CELL TYPE		MECHANISMS AND/OR FUNCTION(S)		PATHWAY	REFERENCES	
*Klebsiella pneumoniae*	Cav-1 KO mice		Cav1 may offer resistance to infection, affecting production of pro-inflammatory cytokines		STAT5 and Akt activity	(76)	
*Salmonella enterica* serovar Typhimurium	Cav-1 KO mice		Cav-1 plays a role in innate immune defense and regulated macrophage cytokine production and signaling		STAT3 activity	(77)	
*Salmonella typhimurium*	HeLa cells		Cav-1 mediates *Salmonella* invasion leading to the formation of membrane ruffles at the apical pole of the host-cell membrane		SopE-dependent Rac1 activation	(78)	
*Pseudomonas aeruginosa*	IB3-1 cells		Cav-1 plays a role in *P. aeruginosa* internalization		–	(79)	
	Cav-1 KO mice		Cav-1 plays a role in innate immune defense and is crucial for resistance to *P. aeruginosa* infection		–	(80)	
*Listeria monocytogenes*	MDCK and HeLa cells (shRNA)		Cav-1 is crucial for efficient bacterial cell-to-cell spreading		–	(81)	
*Trichomonas vaginalis*	HEK-293 cells transfected with caveolin-1		Overexpression of Cav-1 plays a regulatory role during the uptake of extracellular vesicles		–	(82)	
*Escherichia coli*	HEK 293T cells and CSD		CSD peptide induces the activation of immunity		Rab5 activity	(83)	
	hBMEC		Cav-1 plays a significant role in *E. coli* internalization of hBMEC		PKC⍺	(84)	
	Peripheral blood mononuclear cells		Cav-1 plays a potential role during endocytosis		Toll-like receptor 4, Src signalling	(85)	
	hBMECs (shRNA)		Cav-1 plays a role during blood-brain barrier permeability in human endothelial cells		VEGFA signaling cascade	(86)	
HIV-1	hBMEC (siRNA)KO mice		Cav-1 acts as an early and critical modulator in controlling the signaling pathways that lead to the breakdown of tight junction proteins		Ras signaling	(87)	
	Human macrophages		Cav-1 contributes to persistent infection in macrophages and reduction of HIV replication		Tat pathway, p53 activity	(88)	
	Aortic endothelial cell		Regulation of eNOS by Cav-1 and bioactive NO production, can lead to HIV induced pathology	–		(89)
	Human brain pericytes (siRNA)		Cav-1, ocln, and Alix complex affect pro-inflammatory cytokine profile and regulate HIV-1 infection and egress	–		(90)
LPS model		Cav-1 KO mice	Cav-1 regulates the production of eNOS-derived NO and pro-inflammatory proteins, iNOS and ICAM-1	Prevent NF-κB activation		(69)
		Sprague-Dawley rats	Cav-1 plays a role in eNOS induction inhibition under stress	–		(91)
		Murine Macrophages (siRNA)	Cav-1 acts as a potent immunomodulatory effector molecule in immune cells	MKK3/p38 MAPK		(37)
		Cav-1 KO mice	Activation of eNOS secondary to loss of Cav1 induced a dampening of the innate immune response to LPS, attenuating inflammatory lung injury.	Toll-like receptor 4,IRAK4 nitration		(92)
		PMVECs	Cav-1/eNOS signaling pathway has been shown to be involved in the beneficial mechanisms of pravastatin in septic acute lung injury	eNOS signaling pathway		(59)
CLP model		Cav-1 KO mice	Cav-1 attenuates the systemic inflammatory response and protects against septic death. Cav-1 modulates lymphocyte apoptosis and homeostasis in sepsis	–		(66)
		Wistar rats	Cav-1 induces apoptosis and increased necrosis; decreased expression of Cav-1 promoted changes in PAR-1 due to altered occupancy of the EPCR	p38 MAPK kinase		(38)
		C57BL/6 mice	Pravastatin ameliorated acute septic lung injury by suppressing the inflammatory response, apoptosis, and decreasing pulmonary microvascular permeability via regulation of the Cav-1/eNOS signaling pathway	eNOS signaling pathway		(59)

HeLa, Human cervical carcinoma; IB3-1, Bronchial epithelium cells; HEK-29, Human embryonic kidney 29; MDCK, Madin Darby Canine Kidney Cell Line; shRNA. Short hairpin RNA; HEK-293, Human embryonic kidney 293 cells; hBMEC, Human brain microvascular endothelial cell; CSD, Caveolin-1 scaffolding domain; siRNA, Small interfering RNA; LPS, Lipopolysaccharides; CLP, cecal ligation and puncture; PAR-1, protease-activated receptor-1; EPCR, endothelial protein C receptor; PMVECs, Pulmonary microvascular endothelial cells.

Studies using Cav–1 KO mice have linked Cav–1 to innate immunity against several pathogens. Previous study demonstrated that Cav–1 may resist *Klebsiella pneumoniae* infection, the third most microorganism recovered in blood cultures from septic patients, through the actions of STAT5 and the GSK3β–β–catenin–Akt pathway that affected the systemic and local production of pro–inflammatory cytokines (76). Interestingly, Cav–1 KO mice were more susceptible to death after infection with *Salmonella enterica* sv*. typhimurium* (77), suggesting that Cav–1 palys a role in immunity against bacterial pathogens and responds to bacterial infections differently during sepsis. Research carried out in Thailand, Indonesia and Vietnam identified that 2.7% of patients with sepsis were positive for invasive non–typhoidal *Salmonella* (93). In addition, a molecular mechanism for the Cav–1–dependente entry of *Salmonella* into host cells through the regulation of cytoskeletal microfilaments was identified. The bacterial protein SopE was able to interact with Rac1 and regulated actin filaments in epithelial cells during cell invasion. These results described the model of bacterial entry, through the interaction of Cav–1 with SopE and Rac1, promoting an increase in cell ruffling for endocytosis in host cells (78).

Individuals with cystic fibrosis have deficient innate immunity and are susceptible to chronic pulmonary infection by *P. aeruginosa*. This pathogen can be observed in several clinical cases, such as pneumonias, urinary tract infections and sepsis. The epithelial cell response to *P. aeruginosa* infection involved the rapid formation of lipid rafts containing cystic fibrosis transmembrane conductance regulatory protein (CFTR) (94). The efficient internalization of *P. aeruginosa* required CFTR and Cav–1 cluster that was co–localized at sites of attached, invading, and internalized bactéria (79). In addition, Cav–1 promoted bacterial elimination during acute pneumonia and chronic colonization. Absence of Cav–1 reduced PMN recruitment and increased production of inflammatory cytokines during the acute phase of pneumonia, affecting host resistance to infection (80).

*Listeria monocytogenes* can cause several clinical syndromes, most often sepsis, meningitis, and encephalitis, particularly in immunocompromised hosts. The mortality rate is high, reflecting the combination of an immunocompromised individual and an often–late diagnosis. *L. monocytogenes* can trigger Cav–1 recruitment into a neighboring cell with actin filament involvement. Cav–1 endocytosis is the main factor during bacterial cell–to–cell motility, since transfer is significantly blocked in cells Cav–1 depleted (81). Another microorganism such as *Trichomonas vaginalis* related to infection in pregnancy and associated with preterm delivery and postabortion sepsis was internalized in a caveola–dependent way overexpression of Cav–1 played a regulatory role during the uptake of extracellular vesicles (82, 95).

Reported cases of neonatal sepsis from 2015 to 2017 identified ampicillin–resistant *Escherichia coli* infection in 36.6% of infants. Alterations in the distribution and/or resistance to antimicrobials by different pathogens is important prevention strategies that should include efforts to prevent intra–amniotic infection necessary to reduce the incidence of morbidity and mortality from neonatal sepsis (83, 96). Interactions of Cav–1 and protein kinase C were necessary for the intracellular invasion of *E. coli* in human brain microvascular endothelial cells (84). Recent publication demonstrated that *E. coli* endocytosis through Cav–1 scaffolding domain peptide was regulated by GTPase Rab5 in human cells (83). In addition, TLR4 overexpression promoted *E. coli* endocytosis through the caveolae/Cav–1 formation (85). Moreover, Cav–1 played multiple roles in the VEGFA–induced signaling cascade during *E. coli* infection to enhance blood–brain barrier permeability in human endothelial cells (86).

Previous study verified that the leading causes of sepsis and high mortality in sub–Saharan Africa were people with HIV and tuberculosis (97). Authors have provided information on the molecular mechanisms involved in sepsis, brain infection, and neuroinflammatory responses associated with HIV infection, suggesting activation of Ras signaling by twin–arginine translocation (Tat) pathway, upregulation of Cav–1 and disruption of TJ proteins of the blood–brain barrier (87). Subsequently, a study demonstrated a possible mechanism for cross–communication between Tat and p53 in the upregulation of Cav–1 expression causing HIV virus reduction, suggesting that Cav–1 may contribute to persistent infection in macrophages (88). Additionally, HIV virus proteins released from infected endothelial cells in combination with cytokines and chemokines in the extracellular environment can induce changes in Cav–1 distribution and consequently impair endothelial nitric oxide synthase (eNOS) regulation. Moreover, the reduction in eNOS may be related to the eNOS HIV–induced endothelial dysfunction through Cav–1 can be considered as contributors to vascular disease in HIV–positive individuals (89). Furthermore, HIV–1 infection and/or modulation of the Cav–1, occludin and early acting endosomal factor (Alix) complex affected cytokine production by brain pericytes. Modifications of the cav–1, ocln, and Alix complex regulated HIV–1 infection and egress (90).

LPS a component of the outer membrane of Gram–negative bacteria, interacts with specific receptors on the host effector cells and induces the synthesis of many proinflammatory cytokines. The LPS–induced sepsis model has been used in numerous studies due to its similarity to the pathophysiology of severe human sepsis (98). Another model of sepsis, where the cecum is ligated and punctured with a needle was described as a cecal ligation and puncture (CLP) model that results in septic shock with death. The CLP model is associated with polymicrobial sepsis and sepsis. An advantage of this model is that the mortality rate and time to death can be modulated according to the required experimental assay. In this way, the CLP model has become one of the most used intra–abdominal sepsis models in studies of pathophysiology and treatment of abdominal sepsis and its systemic consequences (99). The LPS and CLP models have a similar mortality rate, but differences in the kinetics and magnitude of cytokine production. LPS–induced sepsis does not appear to accurately reproduce the cytokine profile of sepsis (100).

Previous study using LPS–induced sepsis model showed that Cav–1 was able to regulate eNOS–derived NO production and inhibited NF–κB activation and expression of pro–inflammatory proteins, iNOS and ICAM–1 in a mouse model of LPS–induced lung injury (69, 70). In addition, results showed that LPS treatment increased the expression of the CAV–1 protein and the CAV–1/eNOS interaction, demonstrating the main role of Cav–1 in the inhibition of LPS–mediated eNOS activation (91). Cav–1 showed a protective role for inflammation by suppressing the production of pro–inflammatory cytokines (TNF–α and IL–6) and increasing the production of anti–inflammatory cytokines (IL–10) in the LPS model, involving the p38 MAPK signal pathway (37). Evidence of Cav1 modulation on eNOS activity during the regulation of innate immunity and sepsis–induced lung injury has been reported. Activation of eNOS secondary to Cav1 deficiency reduced IRAK4 kinase activity, attenuating TLR signaling mediated by the MyD88–IRAK4 pathway (92). The Cav1–eNOS interaction during sepsis may represent a new therapeutic approach for the treatment of acute lung injury.

Using the CLP model, Cav–1 KO mice were more susceptible to polymicrobial septic death with exaggerated production of proinflammatory cytokines, alteration in lymphocyte homeostasis and high bacterial load in organs such as the liver and spleen (35). A study demonstrated that part of host defense during sepsis is directed towards the control of Cav–1 protein expression in the pulmonary endothelium and regulation of apoptosis through PAR–1/EPCR specificity (38). Thus, the expression of Cav–1 by different mechanisms can modify lung function at a cellular and systemic level, making Cav–1 a promising candidate in the fight against sepsis and mortality. Cav–1 positive cells exhibited reduced protein expression in the septic lung and apoptosis increased significantly after sepsis induction.

## Cav–1 and Apoptosis/Autophagy

Sepsis can induce cellular apoptosis during severe inflammation response. Investigations showed that pulmonary microvascular endothelial cell apoptosis was crucial for cell barrier dysfunction using a model of sepsis–induced lung inflammation (59, 101). Furthermore, suppression of apoptosis has been shown to improve endothelial dysfunction and hyperpermeability in septic mice (59). During sepsis, lymphocytic death by apoptosis represents a major problem (102). Scientific evidence demonstrated that inhibition of apoptosis promoted protection against death from sepsis (103). Inflammatory mediators and other reactive molecules released to eliminate pathogens also cause tissue damage. Cytokines induced by pathogenic microorganisms, toxins and immune mediators promote the stimulation and activation of endothelial cells leading to apoptosis (38, 104).

The role of Cav–1 in the regulation of apoptosis is contestable and appears to be specific to the cell type and stimulus used in the experimental assay. Cav–1 has increased hepatocyte resistance to apoptosis triggered by transforming growth factor–β (6, 105). Conversely, Cav–1 showed no influence on apoptosis in human trabecular meshwork cells (6). Data also showed that Cav–1 KO mice showed increased apoptosis in the thymus and altered T lymphocyte homeostasis during sepsis (74). Cav–1 depletion also mediated protection against apoptosis through the downstream PI3K/Akt and ERK/MAPK pathways, which are favorable for cell survival under stress (106). Moreover, Cav–1 deficiency promoted increased mitochondrial apoptosis, decreased ATP production and inhibition of mitophagy in respiratory epithelium (107). Cav–1 knockdown also blocked cytochrome C release induced by oxidative low–density lipoprotein and caspase 3 activation, in addition to inhibition of NF–κB p65 and EGR1 indicating that Cav–1 was a key mediator for apoptosis in human umbilical vein endothelium cells (108). Thus, studies are essential to clarify the mechanisms underlying cellular apoptosis regulated by Cav–1 and its contribution as a modulator of the inflammatory response during sepsis.

Cav–1 also participates in the activation of autophagy through of the formation of autophagosomes (6, 10, 109, 110). However, the regulatory mechanisms of autophagic flux by Cav–1 during sepsis are not yet known. Autophagy is a cellular defense mechanism used to resist pathogenic microorganisms and dangerous signals, and related to the induction and regulation of the inflammatory immune response in the development of sepsis (111). Autophagy exerts a protective effect on sepsis by eliminating pathogens, neutralizing microbial toxins, and regulating cytokine release (112). Previous study demonstrated that Cav–1 promoted advanced stage autophagy, increasing autophagosome–lysosome fusion (113). In contrast, Cav–1 deficiency also promoted autophagy (114). Additionally, Cav–1 deficiency induced an increase of macrovasculature endothelial cell autophagy. Enhanced autophagy may provide a new mechanism to explain protection against cardiovascular disease progression in the absence of Cav–1 and provide relevant information on the regulation of autophagy by Cav–1 (115). Moreover, high exposure to glucose caused a reduction in TJ proteins mediated by intracellular translocation regulated by Cav–1 and subsequent autophagy, resulting in early disruption of the blood–brain barrier (60). Cav–1 has several multifunctional roles in human diseases, however, there are still many questions of how. Then, regulatory role of Cav–1 in autophagy process should drive research to establish new therapeutic strategies for sepsis treatment.

A novel mechanism of autophagy induced by synthetic iron oxide–based nanoparticles (SPIONs) in mononuclear cells through activation of the Cav1–Notch1/HES1 signal pathway promoted inhibition of inflammation in the sepsis and liver injury model. The results showed that SPIONs have therapeutic potential for the treatment of sepsis (3).

## New Therapies Under Study for the Treatment of Sepsis

The current indicated treatment for sepsis is administration of intravenous antibiotics immediately after the diagnosis of sepsis. Drug therapy should include more than one antimicrobial to ensure broad spectrum to target microorganisms more isolated from sepsis (116, 117). The local epidemiology and antibiotic resistance profile of circulating pathogens is essential for effective treatment. Clinical medicine has achieved important advances with the discovery of biomarkers used for the diagnosis and prognosis of sepsis, however, the disease still has high levels of intensive care unit admission around the world. Importantly, mortality associated with sepsis and septic shock has been decreasing in high–income countries in recent years (118). The increasing number of multidrug–resistant microorganisms represents a serious problem, especially if new drugs are not discovered in the coming years. Thus, the future introduction of new medicines and/or prophylactic measures will play a crucial role in the treatment of sepsis and septic shock (9, 117)

SPIONs have been proposed for use in the diagnosis and treatment of sepsis (118, 119). SPIONs exhibited low cytotoxicity and good biocompatibility, showing significant protective effects against sepsis in preclinical models (120). Cav–1 overexpression was shown to have the ability to inhibit the proliferation of K562 leukemic cells, increase sensitivity to SPIONs and promote autophagy. Consequently, SPIONs participate in the modulation of inflammation and autophagy in macrophages during sepsis, and better knowledge of how this process occurs is important (121).

Another new therapy under study is pravastatin, a competitive inhibitor of 3–hydroxy–3–methyl–glutaryl–coenzyme A (HMG–CoA) reductase, which promoted Cav–1 depletion, preventing caveolar–dependent BK virus (BKV, a human polyomavirus) internalization and repressed BKV infection in human proximal renal tubular epithelial cells (122). Another study showed that pravastatin ameliorated acute septic lung injury by suppressing the inflammatory response, apoptosis, and decreased permeability of lung tissue by regulation of Cav–1/eNOS signaling pathway in cecal ligation *in vivo* and LPS–simulated microvascular lung endothelial cells *in vitro* (59).

*E. coli* is the main cause of bacterial sepsis in high–income countries. Resveratrol was able to inhibit bacterial penetration of the blood–brain barrier, interfering with Cav–1 upregulation and inhibiting the ERK1/2–VEGFA signaling pathway. Furthermore, resveratrol treatment improved survival rate in mice with sepsis and meningitis by decreasing inflammatory cytokines. Thus, resveratrol attenuated septic and meningitic infections induced by *E. coli*, constituting a new approach for prevention and treatment of invasive diseases (86). Moreover, Dexmedetomidine (Dex) is a selective α2–adrenergic receptor agonist that protected against LPS–induced liver injury by inhibiting the NLRP3 signaling pathway and regulating Cav–1 expression during sepsis (123).

Extracelular matrix (ECM) protein is elevated in sepsis patients. Lumican (Lum) comprise a subgroup of secreted ECM proteins that were known as the small leucine–rich repeat proteoglycans (SLRPs). Cav–1 are important to many signaling platforms that may be influenced by ECM proteins. Lum controls receptor traffic by promoting TLR4 and restricting TLR9 during sepsis, through interaction with CD14 and Cav–1 in plasma membrane lipid rafts to promote proinflammatory signals at the cell surface. The protective role for Lum *via* its TLR regulatory functions was observed in human and mouse sepsis (12). These ECM–Cav–1 interactions may also be relevant to pathogens entry and drug–delivery mechanisms, which should be investigated. Another study revealed that treatment using 5–Aza 2–deoxycytidine combined with trichostatin A protected cell barrier integrity through Cav–1 phosphorylation (59, 124). Therefore, elucidation of the role of Cav–1 during sepsis may provide the inclusion of new therapies to treat septic patients. However, the correlation between the results found in cell culture and in KO animals should be translated to human clinical applications.

## Conclusion and Future Perspectives

The present mini–review summarized some regulatory functions, biological roles and mechanisms of Cav–1 in the development of sepsis (10). The understanding of the dynamics and kinetics that govern caveolae/Cav–1 is still limited (125). However, the influence of Cav–1 altering cell permeability during inflammatory processes in sepsis and in the activation of apoptosis/autophagy becomes increasingly evident. In this way, new designs with more precise strategies may contribute with detailed information regarding the cellular functions of Cav–1, as well as the other components of the lipid microdomains, revealing the molecular mechanisms that will allow more efficient therapies for treatment of sepsis and shock septic.

## Author Contributions

PL–C, BP and PN wrote and revised the manuscript. All authors contributed to the article and approved the submitted version.

## Funding

The authors and their work were supported by Fundação de Amparo à Pesquisa do Estado do Rio de Janeiro (FAPERJ) [award number post–doctoral scholarship PDR10 E–26/204.521/2021], Conselho Nacional de Desenvolvimento Científico e Tecnológico (CNPq, Brazil), Sub–Reitoria de Pós–Graduação e Pesquisa da Universidade do Estado do Rio de Janeiro (SR–2/UERJ) and Coordenação de Aperfeiçoamento de Pessoal de Nível Superior – Brasil (CAPES) – Finance Code 001.

## Conflict of Interest

The authors declare that the research was conducted in the absence of any commercial or financial relationships that could be construed as a potential conflict of interest.

## Publisher’s Note

All claims expressed in this article are solely those of the authors and do not necessarily represent those of their affiliated organizations, or those of the publisher, the editors and the reviewers. Any product that may be evaluated in this article, or claim that may be made by its manufacturer, is not guaranteed or endorsed by the publisher.

## Abbreviations

Cav–1, Caveolin–1; NOS, Nitric oxide synthase; TCR, T–cell receptor; eNOS, Endothelial nitric oxide synthase; ROS, Reactive oxygen species; LPS, Lipopolysaccharide; PAR–1, Protease–activated receptor–1; EPCR, Endothelial protein C receptor; TJ, Tight junctions; KO, knockout; siRNA, Small interfering RNA; MMPs, Metalloproteinases; Alix, Early–acting endosomal fator; eNOS, Endothelial nitric oxide synthase; SPIONs, Synthetic iron oxide–based nanoparticles; HMG–CoA, 3–hydroxy–3–methyl–glutaryl–coenzyme A; Dex, Dexmedetomidine; ECM, Extracelular matrix; SLRPs, Small leucine–rich repeat proteoglycans.
